# Performance of Seven SARS-CoV-2 Self-Tests Based on Saliva, Anterior Nasal and Nasopharyngeal Swabs Corrected for Infectiousness in Real-Life Conditions: A Cross-Sectional Test Accuracy Study

**DOI:** 10.3390/diagnostics11091567

**Published:** 2021-08-28

**Authors:** Miroslav Homza, Hana Zelena, Jaroslav Janosek, Hana Tomaskova, Eduard Jezo, Alena Kloudova, Jakub Mrazek, Vera Murinova, Rastislav Madar

**Affiliations:** 1 Hospital Karvina-Raj, Vydmuchov 399, 734 01 Karvina, Czech Republic; mirek.homza@centrum.cz (M.H.); murinova.vera@nspka.cz (V.M.); 2 Department of Internal Medicine, Faculty of Medicine, University of Ostrava, Syllabova 19, 703 00 Ostrava, Czech Republic; 3 Institute of Public Health Ostrava, Partyzánské náměstí 7, 702 00 Ostrava, Czech Republic; hana.tomaskova@zuova.cz (H.T.); eduard.jezo@zuova.cz (E.J.); alena.kloudova@zuova.cz (A.K.); jakub.mrazek@zuova.cz (J.M.); 4 Center for Health Research, Faculty of Medicine, University of Ostrava, Syllabova 19, 703 00 Ostrava, Czech Republic; janosek@correcta.cz; 5 Department of Epidemiology and Public Health, Faculty of Medicine, University of Ostrava, Syllabova 19, 703 00 Ostrava, Czech Republic; rastislav.madar@osu.cz

**Keywords:** COVID-19, SARS-CoV-2, rapid antigen tests, virus culture, saliva, anterior nasal swab, evaluation, qPCR

## Abstract

Many studies reported good performance of nasopharyngeal swab-based antigen tests for detecting SARS-CoV-2-positive individuals; however, studies independently evaluating the quality of antigen tests utilizing anterior nasal swabs or saliva swabs are still rare, although such tests are widely used for mass testing. In our study, sensitivities, specificities and predictive values of seven antigen tests for detection of SARS-CoV-2 (one using nasopharyngeal swabs, two using anterior nasal swabs and four using saliva) were evaluated. In a setting of a high-capacity testing center, nasopharyngeal swabs for quantitative PCR (qPCR) were taken and, at the same time, antigen testing was performed in accordance with manufacturers’ instructions for the respective tests. In samples where qPCR and antigen tests yielded different results, virus culture was performed to evaluate the presence of the viable virus. Sensitivities and specificities of individual tests were calculated using both qPCR and qPCR corrected for viability as the reference. In addition, calculations were also performed for data categorized according to the cycle threshold and symptomatic status. The test using nasopharyngeal swabs yielded the best results (sensitivity of 80.6% relative to PCR and 91.2% when corrected for viability) while none of the remaining tests (anterior nasal swab or saliva-based tests) came even close to the WHO criteria for overall sensitivity. Hence, we advise caution when using antigen tests with alternative sampling methods without independent validation.

## 1. Introduction

The identification of COVID-19 patients as early as possible after their infection is crucial for the successful management of the epidemic. In addition to qPCR, which serves in most countries as a gold standard, rapid antigen tests (RATs) are employed to facilitate early detection of infected patients and their isolation. Numerous studies evaluated various RATs for SARS-CoV-2, with some reporting excellent results meeting or even exceeding the European Center for Disease Control (ECDC)/World Health Organization (WHO) criterion of 80% sensitivity, while others reported poor results. Such studies are reviewed, e.g., in [1,2,3]. Still, the number of studies evaluating individual RATs grows steadily, and some such studies contain a considerable number of evaluated tests (e.g., [4]). Antigen tests are generally considered to be suitable for detecting the most infectious patients, i.e., those at the stage of the onset of symptoms and a few days before/after that. These are the patients with the highest viral loads, which correlates with generally lower cycle thresholds (*C*_t_) in quantitative real-time PCR (qPCR) testing.

Besides tests based on nasopharyngeal swabs (NPS), self-tests (i.e., tests that do not need expertly trained personnel) utilizing other sampling methods have been put into massive use [5]. Such tests use, for example, anterior nasal swabs (ANS) or saliva. In many countries, they are widely utilized, for example, for testing at workplaces or schools as the sampling is much more user-friendly than the collection of nasopharyngeal swabs. However, although quite a few studies on the use of alternate samples for detection of SARS-CoV-2 have been performed (mostly comparing them to NPS in qPCR assays, reviewed, e.g., by Bastos et al. [6]), reports on real-world performances of RAT self-tests using other than nasopharyngeal or oropharyngeal swabs are rare.

In addition, the evaluation of the performance is usually based on a comparison with qPCR and results then to a large degree depend on the test group and, in particular, on the choice of the reference *C*_t_ value (i.e., viral load) for positivity. Low *C*_t_ values below 20 or even 25 will be captured by a majority of tests approved in the EU; however, higher *C*_t_ values, which (i) are in many countries still considered proof of positivity (for example, up to 40 in the Czech Republic) and (ii) which can still identify infectious patients, are detected much less reliably even with NPS-based RATs. This fact is likely often used by manufacturers who claim extremely high sensitivities based on pre-selection of patients with low *C*_t_ values while real-world performances are usually much worse (e.g., [2,7]).

Due to the massive use of the tests with alternate user-friendly sampling methods at schools and occupational settings, we found the lack of independent information on the real-world performances of these tests surprising. Hence, in this study, we aimed to evaluate the performance of six RATs used in the Czech Republic for screening at schools and workplaces and of one test using nasopharyngeal swabs relative to qPCR. We aimed to compare the diagnostic performance of RATs utilizing three sampling methods (NPS–1 test, ANS–2 tests and saliva–4 tests). Moreover, in line with our previously described method [7,8] (see also a brief summary of the reasoning in Discussion), virus culture was performed in all samples where qPCR and RAT results differed to correct the RATs performances on infectiousness.

## 2. Materials and Methods

### 2.1. Patient Group and Sampling

The study was approved by the local Ethics Committee, No. NsPKar/11593/2021. The tests were performed in a setting of a high-capacity COVID testing center during the outbreak in February and March 2021 in Karvina (Czech Republic). All patients coming for the PCR test for SARS-CoV-2 were offered participation in the study. The inclusion criteria were: (i) asymptomatic patients with known contact with a SARS-CoV-2-positive patient or (ii) mildly symptomatic patients with symptoms consistent with COVID-19, as well as (iii) agreement with participation and (iv) signing an informed consent form. In addition, there were exclusion criteria for saliva-based tests, namely eating, drinking, smoking or chewing in the last 10 min to 2 h prior to saliva sampling (see more in the section on antigen testing).

In patients participating in the study, a nasopharyngeal swab was taken by trained medical personnel and placed into 2 mL of the transport medium (D-MEM, 0.5% bovine serum albumin) for qPCR and, if needed, virus culture. The medium was immediately put into a refrigerator operating at 2–4 °C. Sampling for the RAT (always one RAT per patient) was performed in accordance with manufacturers’ instructions; for RATs utilizing ANS or NPS, these swabs were taken by trained personnel from the other nostril than the one for NPS for qPCR, and saliva tests were performed using self-sampling. The antigen test was performed immediately on site, and samples for qPCR were, still cooled, transported to the Public Health Institute Ostrava for analysis and analyzed within 24 h. The PCR sample was also used for viability testing on CV-1 cells (see below in the qPCR and virus culture section). If the cell culture could not be started within 24 h, the samples were frozen at −80 °C and thawed immediately before testing.

### 2.2. Antigen Testing

Seven RAT tests were compared. One of these used nasopharyngeal swabs (NPS) and was included in the battery of tests so that at least one of the more widely accepted RATs using nasopharyngeal swabs was present in the battery of tests. Two tests that are suitable, according to the manufacturers, for use with anterior nasal swabs (ANS) widely used in the Czech Republic were also included in this study. The samples for these three tests were collected by trained medical personnel from the respective part of the nose in accordance with manufacturers’ instructions. The remaining four tests were self-tests based on saliva samples using various methods of sample collection by the tested individuals. Three of the saliva-based tests (tests referred to as Saliva 1, 3 and 4) used a sponge on a stick inserted in the mouth for collection of saliva (“lollipop-tests”) and subsequent extraction of the saliva from the sponge; the test referred to as Saliva 2 was a “spitting” test, i.e., the tested person spit into the provided cup and then pipetted a partial amount of the sample into the buffer. All these tests were also performed in accordance with manufacturers’ instructions. For saliva tests, patients were asked whether they ate/drank/chewed gum/smoked within the last 10 min to 2 h (depending on manufacturers’ instructions). Patients who responded that they did were not tested using the respective saliva test. Nevertheless, we had no way to verify these statements, which remains a limitation of this study (see more in the Discussion).

### 2.3. qPCR and Virus Culture

Direct PCR used the DBdirect COVID-19 Multiplex qPCR Kit (Diana Biotechnologies, Czech Republic) with an automated PCR set up on Agilent Bravo Liquid Handling System. The detection was based on the proof of two SARS-CoV-2 genes, namely genes encoding the spike protein and EndoRNAse. A synthetic internal standard was used for quality control. The overall cycle threshold (*C*_t_) cutoff was 40, and cut-offs for classification into viral load groups were approximately 1.28 × 10^8^ (*C*_t_ = 20), 4 × 10^6^ (*C*_t_ = 25), 1.25 × 10^5^ (*C*_t_ = 30), 3.91 × 10^3^ (*C*_t_ = 35) and 1.22 × 10^2^ (*C*_t_ = 40) RNA copies/mL.

Virus culture using monolayer CV-1 cells (African green monkey kidney fibroblasts) was only performed where the RAT and PCR tests were in disagreement. Cells cultured at 37 °C in Leighton tubes were inoculated with 300 µL of the sample used for the qPCR testing (or blanks) and were microscopically examined, daily, for cytopathic effects of the virus. After 7 days (or once the cytopathic effect was observed in approx. 75% of cells), they were passaged (1:6) and cultured for another 7 days. If no cytopathic effect (i.e., no virus action) was observed over that period, the sample was declared free of viable virus. Where a cytopathic effect was observed, SARS-CoV-2 presence was verified by qPCR. The sensitivity of virus culture method was verified through serial dilution of the virus stock suspension (3 × 10^11^ RNA copies/mL) prepared by culture, both directly and after freezing at −80 °C and thawing. The detection limit of the method in both cases was approx. 10^4^ RNA copies/mL. Within the frame of a previous study [8], we also performed an analysis before and after freezing on 10 real-world samples with cycle thresholds 25–30 (5 samples) and 30–40 (5 samples), with a 100% agreement between results before and after freezing.

### 2.4. Data Analysis

RAT parameters (sensitivity, specificity, positive and negative predictive values) were calculated in Stata v.14 (StataCorp LLC, College Station, TX, USA). As the reference standard, we used both the (i) qPCR result (considered as positive up to *C*_t_ = 40) and (ii) qPCR result corrected for the cell culture in samples where RAT provided different results from PCR (i.e., where qPCR test was positive but no viable virus was detected, the samples were considered negative); see Table 1 for clarification. 95% confidence intervals were calculated for all parameters.

## 3. Results

In all, 2287 samples were taken and analyzed. The numbers of samples analyzed by individual RATs are detailed in Table 2, along with the test parameters calculated relative to the qPCR (positivity threshold of *C*_t_ = 40) and for the same corrected for virus viability, as well as manufacturer-declared sensitivities and specificities (MDSe/Sp). It is obvious that with the exception of the RAT using nasopharyngeal swabs, the performance of none of the tests met the criteria set by WHO and ECDC [1,9]. It also appears that the performance decreases in the order of nasopharyngeal swabs > anterior nasal swabs > saliva swabs, which might be expected (if the seat of infection is in the nasopharynx, it is more likely that virus will be detected there than in the anterior part of the nose or even the mouth). This is true both before and after correction on cell culture results.

After the experience with the first evaluated saliva-based test (Saliva 3), preliminary results were calculated after recruiting approximately 200 patients for Saliva 1 and Saliva 2 tests, and recruitment of patients was stopped at that time as the results were obviously unsatisfactory (sensitivities below 50%). Evaluation of the last test (Saliva 4) was stopped even sooner, after 98 individuals, as it was obvious even then that further continuation of the evaluation would not make much sense (only 1 out of 27 qPCR-positive samples was detected by this test, while two more returned false-positive results).

**Figure 1:**
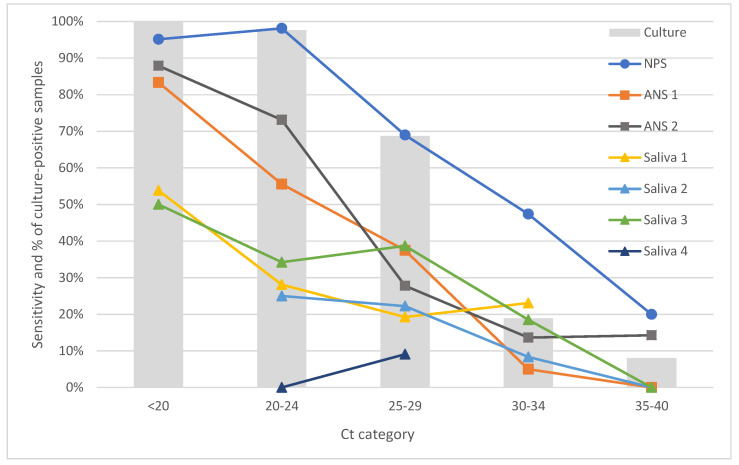
Sensitivities of individual tests calculated relative to qPCR as the gold standard, and presence of viable virus stratified by *C*_t_ cycles; note that cell culture was performed only in 488 samples where qPCR and RAT test results differed; where the respective category included fewer than 5 patients, data are not presented in the graph. NPS–nasopharyngeal swab; ANS–anterior nasal swab; *C*_t_–Cycle threshold.

A closer look at the performance of the tests within individual *C*_t_ categories (see Figure 1) shows that in the *C*_t_ < 20 category, even tests using anterior nasal swabs performed relatively well. However, as soon as in the *C*_t_ < 25 category (*C*_t_ 25 is in most studies evaluating RATs considered as the limit for high positivity due to the original study by Bullard, in which viable virus was not identified in any sample with *C*_t_ > 24 [10]), their performance dropped. All saliva-based tests performed poorly (<60% sensitivity) even in the categories with the highest viral loads (*C*_t_ < 20 and 25, respectively; see Figure 1). Where points are missing in the graph, less than five samples were available in that *C*_t_ category for the particular test, and we decided to remove these points from the presentation.

Percentages of samples falling into individual *C*_t_ categories for individual tests are shown in Table 3.

**Figure 2:**
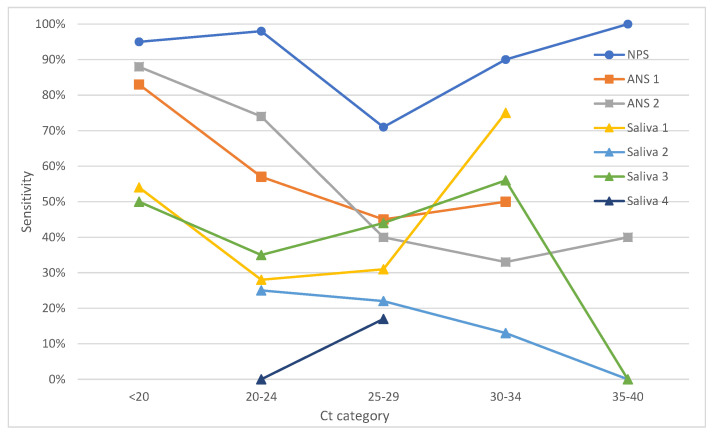
Sensitivities of individual tests with qPCR corrected for the culture results used as the gold standard (see Methods for more information) stratified by *C*_t_ cycles; where the respective category included fewer than 5 patients, data are not presented in the graph. NPS–nasopharyngeal swab; ANS–anterior nasal swab; *C*_t_–Cycle threshold.

Figure 2 shows the same data as Figure 1 after correction on cell culture (i.e., where a qPCR-positive sample contained no viable virus, the sample was considered negative/non-infectious). We can see again that the NPS-based test outperformed all others and, with the exception of the *C*_t_ 25–29 category (which can be probably attributed to the relatively low number of samples in the category—only 28 individuals in this group, resulting in a wide confidence interval of 51–86%), performed well throughout the entire range. The performances of the remaining tests have not improved by much after the correction (Figure 2, Table 2).

Table 4 shows the test performance according to the symptomatic/asymptomatic status of the test subjects. The performance parameters of the NPS test, albeit better for symptomatic individuals, returned a passable result of 84.5% even in the group of asymptomatic patients after the correction on infectiousness. The ANS 1 test showed better sensitivity for symptomatic than asymptomatic individuals, while the results of the ANS 2 did not statistically significantly differ between the symptomatic/asymptomatic individuals after correction on infectiousness; still, the performance failed to meet the WHO/ECDC criteria. The tests using saliva swabs returned unusable results (i.e., far below the WHO/ECDC criteria) with sensitivities of ≤54% in all analyzed classes, regardless of the correction. Still, it should be noted that the number of asymptomatic but SARS-CoV-2 positive individuals was generally low, which is also reflected in the wide confidence intervals in sensitivity and positive predictive values.

Specificities of the NPS and ANS tests were relatively good, with ANS 2 just failing (97.4%) to meet the sensitivity criteria set by WHO and the remaining two (ANS 1 and NPS) meeting the criteria (Table 2).

## 4. Discussion

In this study, we have evaluated the real-world performances of seven RATs in a setting of a high-capacity testing center using our previously proposed method [7,8]. Briefly, the reasoning is that the principal aim of RATs is to identify infectious patients, while PCR can detect dead viral particles that may have been excreted from the organism during recovery, killed by good mucosal immunity or even got to the nasal mucosa already dead (e.g., on dust particles). This can be partially offset by reducing the C_t_ threshold (typically to C_t_ 25); however, viable virus can be detected even at higher C_t_ values. Therefore, verifying the tests only against PCR can lead to overestimating (if C_t_ threshold is reduced) or underestimating (if any PCR positivity even at high C_t_ values is considered) the test sensitivity for the identification of infectious individuals. Virus culture (i.e., virus viability testing) is a possible solution to this problem. Viability testing is, however, extremely laborious and time consuming and for this reason, the approach of viability testing serving only as a “referee” for samples where the two methods disagree is, in our opinion, the best practically achievable solution.

Tests using other sampling methods than NPS were highly inferior to the NPS-based test, the performance of which meets the WHO/ECDC criteria and is in line with the better tests evaluated in our previous study [7]. This does not necessarily mean that all self-tests using saliva or nasal swabs are so vastly inferior, but the fact that all these tests failed definitely makes one doubt the effectiveness of these tests in the high-capacity setting in general.

As expected, the RATs performed the best at the lowest C_t_ cycles, which are associated with a higher viral load and, thus, with higher probability of triggering the test reaction. Below C_t_ 20 (i.e., 1.28 × 10^8^ RNA copies/mL sample), tests using NPS as well as ANS had over 80% sensitivity, thus meeting the ECDC/WHO criterion for sensitivity. However, as soon as in the next category, i.e., C_t_ < 25, where the virus culture confirmed the presence of viable virus (i.e., infectiousness) in almost all samples, only the NPS test maintained a good sensitivity of well over 95%; the result dropped to 73% and 56% for the two ANS tests, respectively. Tests using saliva failed to produce meaningful results even in the categories with the strongest positivity. It is necessary to say that there were very few SARS-CoV-2-positive individuals in the C_t_ < 20 group when testing two of the saliva-based tests (four and two samples, respectively), so the results of the evaluation of these tests in this category are not very reliable; nevertheless, the fact alone that none of these six strongly positive patients were detected supports the conclusion that the performance of these tests is as poor in this category as it is in the others.

Above C_t_ 25, the performance of all tests continued to drop, which was, however, at the same time, accompanied by a drop in the percentage of samples with viable virus. Here, we have to point out that we have detected viable virus in 20% of samples with C_t_ between 30 and 35, and surprisingly, even in 8% of tested samples with C_t_ between 35 and 40 (please note that only 488 samples with discrepant results between RAT and qPCR were analyzed using cell culture). This supports our opinion voiced in our previous papers [7,8] that simply reducing the C^t^ threshold for classifying patients as positive (i.e., the method obviously often employed by the manufacturers when performing their validation studies) is not the way to go for validation. This is also supported by the study on the relationship between virus viability and C^t^ threshold/number of RNA copies in the sample by La Scola [11] who found viable virus up to C_t_ threshold 33 (interestingly, as much as 50% of samples at C_t_ 32 contained viable virus). It is true that they found no viable virus at C_t_ values over 33, but they tested only one to three samples at these thresholds, so their failure to detect any viable sample at these C_t_ values is not surprising (in our study, 90 samples with C_t_ 30–34 and 62 samples with C_t_ 30–35 were tested, respectively, thus giving us a chance to capture even lower percentages of samples with viable virus). The same can be said about the results by Bullard who, as mentioned above, did not detect any viable virus in samples with C_t_ > 24; nevertheless, it is not clear from their paper how many samples fell within the category of C_t_ > 25 (their study included 90 samples in total, with median C_t_ 23) [10].

Comparison with the literature is difficult. We have not found any peer-reviewed paper evaluating saliva-based RATs and even studies using anterior nasal swabs are relatively rare. In one of the few such studies, Osmanodja et al. [12] described an excellent performance of their antigen test using anterior nasal swabs (Dräger Antigen Test SARS-CoV-2), with an overall sensitivity of 88.6% (and as much as 96.7% for patients with a high or medium viral load corresponding to C_t_ = 27 in our study). The nasal swab variant of one of the most popular NPS tests, Standard Q, was evaluated by Nikolai et al. [13]. In their study, professionally taken ANS were compared with PCR and so were self-sampled and professionally collected mid-turbinate swabs. Their results returned excellent performance (86% sensitivity) of this test for professional ANS up to the virus load equivalent to approx. C_t_ 24 in our study, dropping to 43% for higher values. Another ANS-based RAT by a recognized producer, Abbott BinaxNOW™, was evaluated by Pollock et al., reporting an overall sensitivity of 81.2% for patients with C_t_ values up to 35 in self-collected ANS [14] and by Pilarowski et al., reporting 93.3% sensitivity in professionally-collected ANS [15]. These results were, however, in contrast with those by James et al. [16] who reported sensitivity of only 51.6% in professionally-collected nasal swabs using this test.

The two tests using anterior nasal swabs in our study did not perform as well as those reported in the aforementioned studies, suggesting their inferior quality. It must be also noted that both these ANS tests can, according to the manufacturers’ instructions, be used with NPS instead. It is likely that if professionally-collected NPS were used with these two tests instead, the results would be better than those presented in this study. We have, however, not performed such a direct comparison, as the principal reason for the wide use of these tests in the Czech Republic is their “user-friendliness“, i.e., the fact that they do not need a (professional) NPS taken.

None of the saliva-based tests yielded results that could justify their use in practice. We have to acknowledge as a limitation of the study that we do not know whether the patients told the truth that they have not eaten or drunk for some time before the sampling. Nevertheless, from the perspective of the mass use of these self-tests at workplaces, at schools or at high-capacity testing points, a limitation such as not eating, drinking, chewing, smoking, brushing teeth or generally interfering with the oral cavity for 2 h prior to taking the test would render such a test unsuitable for large-scale use regardless of the test result (although 30 min required by some of the tests is perhaps achievable). Their use as self-tests in the morning upon waking, i.e., after a long period without interference with the oral cavity, could perhaps provide better results; nevertheless, in our high-throughput setting, the performance of saliva-based RATs was sadly lacking.

The evaluation of the results for asymptomatic patients is, in view of their sensitivity results, meaningless for the saliva-based tests. For ANT and NPS tests, the results were affected by a low number of positive results in this group, which leads to wide confidence intervals. In effect, we cannot make any strong statements regarding the performance of the tests in these groups and the results can be only perceived as indicative. Still, it appears that the sensitivity is somewhat lower in asymptomatic individuals than in symptomatic ones. This is logical since in symptomatic individuals who are sneezing, the virus is more likely to reach the lower levels of the nasal system. The good news from the perspective of usability of these tests is that in the NPS-based test, the sensitivity estimate remained over 80% even in asymptomatic patients.

Based on our results, we have to strongly disagree with the widespread policy that all CE-certified antigen tests have the same validity, and this is especially true of the tests that have not been independently evaluated. All tests evaluated in our study were CE-certified and their reported sensitivities were over 89%, most of them over 95%, thus allegedly meeting the criteria set by ECDC and WHO. Only the NPS test met the criteria with its 91.2% sensitivity, 98.5% specificity, 96.2% PPV and 96.6% NPV after correction for the presence of viable virus, which is (i) in accordance with the declared values and (ii) comparable to the better performing NPS RATs evaluated using the same method; see our previous work comparing five NPS tests [8]. In that study, some tests provided excellent results (up to >96% sensitivity when compared with PCR and corrected for viability), while others failed to meet the criteria. This variability within the same sampling design also means that well-performing tests from saliva or ANT can exist; in our study, however, none of such tests met the WHO criteria when used in a high capacity setting. It is also worth noting that one of the saliva-based tests that performed poorly in this study is produced by the same manufacturer as an NPS test performing very well in the previous study [8]. This only further supports the notion that poor performance of saliva-based tests is rather associated with the type of the sample than with a poor manufacturing process.

As with every study, ours comes with strengths and limitations. The strengths include a large number of tests, sufficient numbers of tested individuals and, in particular, the cell culture testing for excluding samples that were qPCR-positive but did not contain any viable virus. Besides the aforementioned limitation for saliva-based tests (no way of knowing whether the patients told the truth about not eating, etc., within a specified period before sampling), there is another important limitation to our study. We are, unfortunately, unable to disclose the manufacturers or the test names as the study was funded by distributors who gave their consent for publishing the results only providing that the tests are not named. We acknowledge this as a limitation of this study; however, without this support, this study would not even come to existence. For this reason, we would like to call for additional fully independent studies evaluating the diagnostic performance of, in particular, ANS and saliva-based tests.

## 5. Conclusions

Of the evaluated antigen tests, only the test using nasopharyngeal swabs met the criterion of >80% sensitivity in a real-world high throughput setting in a high prevalence population (consisting of individuals with symptoms or with a history of contact with a SARS-CoV-2-positive person). The evaluated antigen tests using anterior nasal swabs performed much worse, meeting the criterion of 80% sensitivity only in patients with the highest viral load (C_t_ < 20) but dropping below the limit as soon as in the C_t_ 20–25 category. The tests utilizing saliva included in this study yielded the worst sensitivities, with the best of these tests returning sensitivity of 54% in the group with the highest viral load (C_t_ < 20) despite high declared sensitivity values. For these reasons, we strongly caution against using RATs, especially anterior nasal swab-based or saliva-based RATs, solely on the basis of manufacturer-declared sensitivity and specificity values without independent validation. We would also like to use this opportunity to call for such independent studies evaluating the diagnostic performance of, in particular, swab- or saliva-based RATs.

## Figures and Tables

**Table 1:** Explanation of the calculation of test result values without and with correction on viability; + = positive; − = negative.

	Relative to qPCR	Relative to qPCR Corrected for Viability
True positives (TP)	PCR+/RAT+	PCR+/RAT+; PCR− *or* viability+/RAT+
True negatives (TN)	PCR−/RAT−	PCR−/AT− *or* PCR+/viability−/AT−
False positives (FP)	PCR−/RAT+	PCR−/viability−/AT+
False negatives (FN)	PCR+/RAT−	PCR+/viability+/AT−

**Table 2:** Overall real-world performances of 7 RATs; note that only samples where results of RAT and qPCR differed were cell-culture tested; *C*_t_-threshold for qPCR positivity is 40; values are presented as parameter estimate (95% confidence interval); MDSe/Sp = manufacturer-declared sensitivity/specificity; NPS–nasopharyngeal swab; ANS–anterior nasal swab; NPV/PPV–negative/positive predictive value; N–number of subjects.

Test	Sample Type	NPS	ANS 1	ANS 2	Saliva 1	Saliva 2	Saliva 3	Saliva 4
	*MDSe/Sp*	*>93/100*	*>98/>99*	*>95/>99*	*>95/>98*	*>92/>99*	*>89/>96*	*>98/>99*
	*N of Patients*	*480*	*488*	*406*	*217*	*191*	*407*	*98*
**Uncorrected for cell culture**	*Prevalence*	*32.30%*	*26.40%*	*42.40%*	*43.30%*	*41.90%*	*42.80%*	*29.0%*
		*(28.1–36.7%)*	*(22.60–30.60%)*	*(37.5–47.3%)*	*(36.6–50.2%)*	*(34.8–49.2%)*	*(37.9–47.7%)*	*(20.0–38.6%)*
	**Sensitivity**	**80.60%**	**46.50%**	**54.10%**	**25.50%**	**15.00%**	**32.80%**	**3.60%**
		(73.5–86.5%)	(37.70–55.50%)	(46.3–61.7%)	(17.1–35.6%)	(8.0–24.7%)	(25.8–40.3%)	(0.10–18.3%)
	**Specificity**	**98.50%**	**99.40%**	**97.40%**	**98.40%**	**89.20%**	**89.30%**	**97.10%**
		(96.4–99.5%)	(98.00–99.90%)	(94.5–99.1%)	(94.2–99.8%)	(81.9–94.3%)	(84.6–92.9%)	(90.1–99.7%)
	PPV	96.20%	96.80%	93.90%	92.30%	50.00%	69.50%	33.30%
		(91.3–98.7%)	(88.80–99.60%)	(87.3–97.7%)	(74.9–99.1%)	(29.1–70.9%)	(58.4–79.2%)	(0.80–90.60%)
	NPV	91.4%	83.80%	74.30%	63.40%	59.30%	64.00%	71.60%
		(88.0–94.1%)	(80.00–87.20%)	(69.0–79.1%)	(56.1–70.2%)	(51.4–66.8%)	(58.5–69.2%)	(61.40–80.40%)
N of cell culture tested samples (viable virus present/absent)		*35 (11/24)*	*69 (37/32)*	*78 (28/40)*	*72 (42/30)*	*66 (39/27)*	*141 (81/60)*	*27 (16/11*)
**Corrected for cell culture**	*Prevalence*	*28.50%*	*20.30%*	*34.00%*	*30.40%*	*33.00%*	*34.20%*	*19.0%*
		*(24.5–32.8%)*	*(16.80–24.10%)*	*(29.4–38.8%)*	*(24.4–37.0%)*	*(26.4–40.1%)*	*(29.6–39.0%)*	*(12.0–28.6%)*
	**Sensitivity**	**91.20%**	**60.60%**	**67.40%**	**36.40%**	**19.00%**	**41.00%**	**5.30%**
		(85.2–95.4%)	(50.30–70.30%)	(58.9–75.1%)	(24.9–49.1%)	(10.2–30.9%)	(32.7–49.7%)	(0.1–26.0%)
	**Specificity**	**98.50%**	**99.50%**	**97.80%**	**98.70%**	**90.60%**	**90.70%**	**97.50%**
		(96.6–99.5%)	(98.20–99.90%)	(95.2–99.2%)	(95.3–99.8%)	(84.2–95.1%)	(86.5–93.9%)	(91.2–99.7%)
	PPV	96.20%	96.80%	93.90%	92.30%	50.00%	69.50%	33.30%
		(91.3–98.7%)	(88.80–99.60%)	(87.3–97.7%)	(74.9–99.1%)	(29.1–70.9%)	(58.4–79.2%)	(0.8–90.6%)
	NPV	96.60%	90.80%	85.30%	78.00%	69.50%	74.80%	81.1%
		(94.1–98.2%)	(87.70–93.40%)	(80.9–89.1%)	(71.5–83.7%)	(61.9–76.3%)	(69.7–79.4%)	(71.7–88.4%)

**Table 3:** Percentages of samples falling into respective *C*_t_ categories for individual tests.

	1. <20	2. 20–24	3. 25–29	4. 30–34	5. 35–40
NPS	26.97	34.87	19.08	12.50	6.58
ANS 1	18.60	41.86	18.60	15.50	5.43
ANS 2	19.19	38.95	20.93	12.79	8.14
Saliva 1	13.98	34.41	27.96	13.98	9.68
Saliva 2	2.60	25.97	35.06	15.58	20.78
Saliva 3	17.24	41.95	17.82	15.52	7.47
Saliva 4	14.29	32.14	39.29	7.14	7.14

**Table 4:** Comparison of the test parameters presented separately for symptomatic and asymptomatic individuals both before and after correction on virus culture. Test parameters are presented as estimates with confidence intervals in brackets and italics. NPS–nasopharyngeal swab; ANS–anterior nasal swab; NPV/PPV–negative/positive predictive value; N–number of subjects.

		Symptomatic						Asymptomatic					
Test (Sample Type)		N	Prevalence	Sensitivity	Specificity	PPV	NPV	N	Prevalence	Sensitivity	Specificity	PPV	NPV
**NPS**	PCR	237	49.0 *(42.4–55.5)*	**84.5** *(76.6–90.5)*	99.2 *(95.5–100)*	99.0 *(94.5–100)*	87.0 *(80.2–92.1*)	227	15.0 *(10.6–20.3)*	**64.7** *(46.5–80.3)*	97.9 *(94.8–99.4)*	84.6 *(65.1–95.6)*	94 *(89.8–96.9)*
	PCR+viab.		44.3 *(37.9–50.9)*	**93.3** *(86.7–97.3)*	99.2 *(95.9–100*)	99.0 *(94.5–100)*	94.9 *(89.8–97.9)*		11.9 *(8.0–16.8*)	**81.5** *(61.9–93.7)*	98.0 *(95.0–99.5)*	84.6 *(65.1–95.6)*	97.5 *(94.3–99.2)*
**ANS 1**	PCR	194	48.5 *(41.2–55.7)*	**52.1** *(41.6–62.5)*	99.0 *(94.6–100*)	98.0 *(89.4–100)*	68.8 *(60.5–76.2)*	288	11.5 *(8–15.7)*	**30.3** *(15.6–48.7)*	99.6 *(97.8–100)*	90.9 *(58.7–99.8)*	91.7 (*87.8–94.7)*
	PCR+viab.		37.6 *(30.8–44.9)*	**67.1** *(55.1–77.7)*	99.2 (*95.5–100)*	98.0 *(89.4–99.9)*	83.3 *(76.2–89.0)*		8.3 *(5.4–12.1)*	**41.7** *(22.1–63.4)*	99.6 *(97.9–1)*	90.9 *(58.7–99.8)*	94.9 (*91.7–97.2)*
**ANS 2**	PCR	243	60.5 *(54–66.7)*	**56.5** *(48.1–64.6)*	96.9 *(91.1–99.4)*	96.5 *(90.1–99.3)*	59.2 *(51.1–67)*	158	14.6 *(9.5–21.0)*	**43.5** *(23.2–65.5)*	97.8 *(93.6–99.5)*	76.9 *(46.2–95)*	91 *(85.2–95.1)*
	PCR+viab.		50.6 *(44.2–57.1)*	**67.5** *(58.4–75.6)*	97.5 *(92.9–99.5)*	96.5 *(90.1–99.3)*	74.5 *(67.0–81.1)*		9.5 *(5.4–15.2)*	**66.7** *(38.4–88.2)*	97.9 *(94.0–99.6)*	76.9 *(46.2–95.0)*	96.6 *(92.1–98.9)*
**Saliva 1**	PCR	127	55.1 *(46.0–64.0)*	**27.1** *(17.2–39.1)*	96.5 *(87.9–99.6)*	90.5 *(69.6–98.8)*	51.9 *(42–61.7)*	86	25.0 *(16.2–35.6)*	**23.8** *(8.2–47.2)*	100 *(94.3–100)*	100 *(47.8–100)*	79.8 *(69.2–88)*
	PCR+viab.		38.6 *(30.1–47.6)*	**38.8** *(25.2–53.8)*	97.4 *(91.0–99.7)*	90.5 *(69.6–98.8)*	71.7 *(62.1–80.0)*		16.7 *(9.4–26.4)*	**35.7** *(12.8–64.9)*	100 *(94.9–100)*	100 *(47.8–100)*	88.6 *(79.5–94.7)*
**Saliva 2**	PCR	116	60.3 *(50.8–69.3)*	**15.7** *(8.1–26.4)*	84.8 *(71.1–93.7)*	61.1 *(35.8–82.7)*	39.8 *(30–50.2)*	75	13.3 *(6.6–23.2)*	**10.0** *(0.3–44.5)*	92.3 *(83–97.5)*	16.7 *(0.4–64.1)*	87 *(76.7–93.9)*
	PCR+viab.		49.1 *(39.7–58.6)*	**19.3** *(10.0–31.9)*	88.1 *(77.1–95.1)*	61.1 *(35.7–82.7)*	53.1 *(42.7–63.2)*		8.0 *(3.0–16.6)*	**16.7** *(0.4–64.1)*	92.8 *(83.9–97.6)*	16.7 *(0.4–64.1)*	92.8 *(83.9–97.6)*
**Saliva 3**	PCR	219	60.7 *(53.9–67.2)*	**36.1** *(28–44.9)*	93.0 *(85.4–97.4)*	88.9 *(77.4–95.8)*	48.5 *(40.6–56.4)*	182	20.3 *(14.7–26.9)*	**21.6** *(9.8–38.2)*	87.6 *(81.1–92.5)*	30.8 *(14.3–51.8)*	81.4 *(74.4–87.2)*
	PCR+viab.		50.7 *(43.9–57.5)*	**43.2** *(33.9–53.0)*	94.4 *(88.3–97.9)*	88.9 *(77.4–95.8)*	61.8 *(53.9–69.3)*		13.2 *(8.6–19.0)*	**33.3** *(15.6–55.3)*	88.6 *(82.6–93.1)*	30.8 *(14.3–51.8)*	89.7 *(83.9–94.0)*
**Saliva 4**	PCR	37	46.0 *(29.5–63.1)*	**5.9** *(0.2–28.7)*	100 *(83.2–100)*	100 *(2.5–100)*	55.6 *(38.1–72.1)*	57	17.5 *(8.8–29.9)*	**0** *(0–30.9)*	95.7 *(85.5–99.5)*	0 *(0–84.2)*	81.8 *(69.1–90.9)*
	PCR+viab.		32.0 *(18.0–49.8)*	**8.3** *(0.2–38.5)*	100 *(86.3–100)*	100 *(2.5–100)*	69.4 *(51.9–83.7)*		11.0 *(4.0–21.5)*	**0.0** *(0.0–45.9)*	96.1 *(86.5–99.5)*	0.0 *(0.0–84.2)*	89.1 *(77.8–95.9)*

## Data Availability

Data are available from the authors upon reasonable request.
